# COVID-19: Another Look at Solidarity

**DOI:** 10.1017/S0963180120001115

**Published:** 2020-12-21

**Authors:** Matti Häyry

**Affiliations:** Aalto University School of Business

**Keywords:** COVID-19, solidarity, corona, justice, equality, copathy

## Abstract

Is there such a thing as corona solidarity? Does voluntary mutual aid solve the problems caused by COVID-19? I argue that the answer to the first question is “yes” and to the second “no.” Not that the answer to the second question could not, in an ideal world, be “yes,” too. It is just that in this world of global capitalism and everybody looking out for themselves, the kind of communal warmth celebrated by the media either does not actually exist or is too weak to rule out the uglier manifestations of group togetherness, driven partly by the pandemic. I make my point by offering two approaches to understanding what solidarity is. According to the first, it is essentially partiality: “us” against “them.” According to the second, it can be many things, including the impartial promotion of the good of others. I show that the second reading would make it possible for mutual aid to solve the problems caused by COVID-19 and other crises. This would happen at the expense of conceptual clarity, but that is a minor concern. The major concern is that the more natural manifestations of group togetherness are incited by negative feelings. This is par for the course within the narrower reading of solidarity, but it means that the potentially positive ideas of identity, care, communal values, and special relations are displayed in violent confrontation instead of a calm recognition of the threats that most of us face together.

Solidarity has become an ethical buzzword during the 2020 corona pandemic. A popular theme in the media from early on has been that people and countries have begun to take care of one another, and a universal feeling of togetherness has washed away interest conflicts and ideological disagreements. Russia sends experts to Italy, China advices the rest of the world on how to control the pandemic, Cuba sends medical teams abroad to help in the fight, European Union (EU) countries agree on significant mutual economic aid, and all humankind has a common sense of purpose in its fight against the coronavirus disease 2019 (COVID-19), caused by severe acute respiratory syndrome coronavirus 2.^1^^,^^2^

Since this sounds too good to be true, it probably is. But how and to what extent? In the following, I will take a closer look at the situation. I will start by defining solidarity narrowly, in a way that distinguishes it from neighboring psychological, moral, and political mechanisms and principles. I will then present the ways in which nations and individuals have expressed their willingness to help others; and show that very few of them match my narrow definition. I will go on to give six wider definitions of solidarity, based on my previous studies on equality and justice; and show how the reported forms of mutual support either still fail to register as solidarity or get the label on strained conceptual grounds. To conclude, I will return to the narrow definition and suggest that the most characteristic examples of “corona solidarity” are not of the warm and cuddly we are all in this together kind, but demonstrations of identity and exclusion.

## Defining Solidarity Narrowly

Solidarity means feeling and acting together in the pursuit of a goal shared by a group with common interests or beliefs. It bears resemblances to sympathy, altruism, and justice, but differs from these, according to a narrow account, by being automatically and necessarily partial.^3^^,^^4^ I and my siblings against our cousins; I, my siblings, and our cousins against the rest of the village; I, my siblings, our cousins, and the rest of the village against the rival village; and so on. The limit of solidarity—how far it can be extended—is a matter of definition, but to stand out in the crowd, the principle has to set “us” against a defined group of “them.”

Other features of solidarity, when it is seen as an alternative to the individualistic bioethical notions of (personal) autonomy, (contractual) justice, and (calculable) utility, include that it is communal, voluntary, spontaneous, and reciprocal, as opposed to state-controlled, externally enforced, organised, and contract-based (see note 4).

Solidarity is not, then, psychological benevolence, or *sympathy*, offered as a behavior-explaining mechanism by Scottish Enlightenment philosophers. David Hume and Adam Smith believed that when we see others suffer, the suffering resonates in us, albeit in a milder form, and makes us want to stop it or eliminate its cause. This is how many people see solidarity, but if we take the partiality clause seriously, this understanding is not terminologically helpful. Unlike solidarity (in the narrow sense), sympathy can extend to all human (and sentient nonhuman) beings.^5^

It is not moral altruism, either. By moral altruism I mean the internalized obligation to further other people’s interests as well as one’s own. Leaning away from egoism and toward helping others, without expectations of an immediate reward, is a part of most ethical stances, be their foundation in religious teachings or philosophical theories. Altruism may or may not be partial, and solidarity can be compatible with its selective versions. It is not, however, in line with unconstrained altruism, due to its inherent partiality.

Nor is it akin to political justice, except in one specific sense. If we believe in collectivist and communal values, different rights and duties to different kinds of people, and the primacy of “our” interests over the interests of “others” or “all,” solidarity is our natural interpretation of justice.^6^ In this case, however, we can expect to be challenged by those who hold that justice is individual-centered, universalist, and altruistic. For the sake of conceptual clarity, it is probably sensible to keep solidarity and justice apart, when possible.

At its purest, solidarity means protecting one’s *lifeworld* (Lebenswelt). A lifeworld can include an awareness of the interests of oneself and others included in one’s sphere, but its main content is immaterial, probably cultural, or spiritual. At its most genuine, solidarity is also spontaneously grown, although the realization of its existence can be induced, like the class consciousness of workers according to Karl Marx. It is not, however, primarily a voluntary contract or association between individuals, although some welfare-state type social insurance systems are confusingly called solidaristic^7^; nor is it a dole given by those further up on a social scale to those further down on the same scale, although something like this has been recently proposed.^8^

Solidarity can, following socialist parlance, be effortlessly extended to the workers of the world, the exploited, the oppressed, and the precariat. Even in these cases, however, an adversary remains, usually global capitalism in its corporate or state disguises. The role of “them” is in these scenarios reserved to those who benefit from the arrangement, in other words, to the extremely well to do (who almost always benefit) and to the moderately well to do (who sometimes benefit). Extended political solidarity may or may not come with an anticipation of universal bliss once detrimental power structures have been eliminated. If it does, then this aspiration turns solidarity into an instrument of promoting universal justice, which is a more general goal. Solidarity in its more confined sense must enter, again, a competition between all other versions of justice that pursue the same end and claim to be instrumental in its achievement.

There are other accounts of the concept, and I will get to those in due course, but first let me present some typical examples of (alleged) corona solidarity and demonstrate how they fail to live up to the expectations placed on them.

## Acts of Kindness and Public Relations Maintenance

Already during the early weeks of the COVID-19 pandemic, media outlets began to hail private and public acts of kindness and mutual support, which some of them dubbed “corona solidarity” (see notes 1 and 2).

Individual citizens volunteered to assist their neighbors by shopping or walking dogs, residents in isolation started to sing together on their balconies to keep their spirits up, half a million people in the United Kingdom offered their time to help the National Health Service (NHS), and large gatherings applauded nurses for their selfless dedication. As commendable as these activities may be, insofar as they are intended to promote the interests of others, they fall under the categories of sympathy and altruism rather than group-focused solidarity. The ‘we’ feeling that I may have with those in need of help, and with healthcare professionals, is based on our shared humanity, nothing more specific. Besides, set in their wider political context, many of these undertakings cease to look unequivocally desirable. The people who volunteered to help in the NHS are not to blame, but when the government of an alleged welfare state tries to pass the burden of health services to individual citizens, something is not quite right. In a related vein, nurses and their labor unions have noted that heart-warming as the accolade may be, they would have preferred better working conditions and decent salaries to hand clapping in the streets.

Another strand of tales on early corona solidarity paraded businesspeople reaching out to their communities. Artists began to live stream free performances. Local businesses provided special delivery services for people in isolation. Grocery stores opened an hour earlier to cater to the needs of those who cannot shop at the same time as others. Multinational restaurant chains announced that their operations will undergo changes to protect their customers and employees.

Some good has, no doubt, been done by these innovations. Here again, however, sympathy and altruism seem to be more prominent than solidarity. Our attention is drawn to the natural or moral goodness of these actors, not to interests or values that they might share with their clientele.

Even the role of benevolence can be contested in this second patch of contestants for corona solidarity. Artists are trying to make a living, and especially in difficult times this does mean sharing the fruits of their talent without compensation. Delivery services invigorate businesses that would otherwise suffer, so the motive for their existence is self-interest. As a negative bonus, the home delivery system, which was exploitative to begin with, became, due to competition, even more so.^9^ Regarding the extra opening times of grocery stores, I do not believe that my mother, 89, and father, 91, are alone in observing, “Who in their right mind is up, let alone seriously purchasing goods, in the wee hours of the morning?” Whatever fellow feeling prompted this practice was not, then, the safest universal guide to go by.

Multinational corporations are in a league of their own when it comes to spinning governmental regulations to expressions of concern for social responsibility. As an example, it took Starbucks only 2 days to change their message from “[we] are taking guidance from the CDC [Centers for Disease Control and Prevention] and local health authorities” to “we remain committed to honest conversation, taking care of one another, and making decisions with you in mind—always through the lens of Our Mission and Values”^10^ when the first public restrictions hit them in March.^11^ The Public Relations Department swiftly erased the reactive “we are following orders” communication and substituted a proactive “we are here for you and our joint values” message. A linguistic turn toward solidarity, but no change in the substance. The workers and customers are still exposed to contagion; words do not alter that.

Governments are looking after their own interests even more blatantly than businesses. Russia’s offer to help Italy was a public relations stunt performed mainly for the home audience,^12^ China has sent teams all over the world to polish their brand, stained by the origin of the COVID-19 pandemic in Wuhan,^13^ and Cuba’s international medical teams have for long been the country’s ticket to gain sympathy for their regime, otherwise frowned upon.^14^ The EU did reach a consensus on a coronavirus recovery fund, but the result was bitterly contested and the leaders returned from the summit already planning how they could benefit from the deal at the expense of others.^15^

## Defining Solidarity Broadly—Helpful But Not *That* Helpful

Many activities suggested as instances of corona solidarity do not make the cut, if the narrow definition is used. Since journalists have, nevertheless, been able to formulate a coherent story and to sell it to their readers, a different take on solidarity must be more popular. Let me show how the concept can have six very different meanings, if it is observed through the theoretical lenses of equality and justice. The connection is based on the fact that the three principles are almost indistinguishable in many accounts of healthcare and welfare (see note 7).^16^

**Figure 1:**
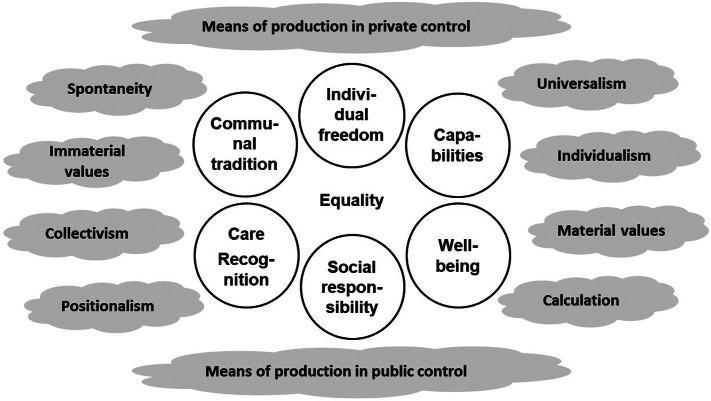
Values and beliefs on a map of justice as interpretations of equality.

Figure 1 presents a map showing the positions of some of the main theories of justice as interpretations of equality and in the context of the beliefs and values associated with them (see note 6).

Almost everyone believes, in some sense, in the importance of equality, in the middle of Figure 1. Different values and beliefs, however, draw interpretations of justice into different directions. The three main axes in the figure are private versus public control of means of production (top–bottom), local versus global interests (top left–bottom right), and positional versus universal norms and values (bottom left–top right). The theories most naturally located in the six spheres on the map are, from the top and proceeding counterclockwise, libertarianism, communitarianism, care and recognition ethics, socialism, utilitarianism, and the capability approach (see note 6).

On the left in the map, intrinsic values include spontaneous communality, immaterial values, collectivism, and significant ethical differences between groups or sections of people. On the right, the commitment is to the similar moral standing of all people (and maybe other sentient beings), autonomous choices by individuals, measurable goods, and the meticulous calculation and weighing of these goods as the basis of public decisions. The emphasis on private property or particular communities and sections renders the upper left side slightly egoistic. On the lower right, altruism has a firmer grip, as the creeds there stress universal inclusion, social responsibility, and equality of opportunity for all. The egoism-altruism distinction is not, however, either exact or rigid.^17^

**Figure 2:**
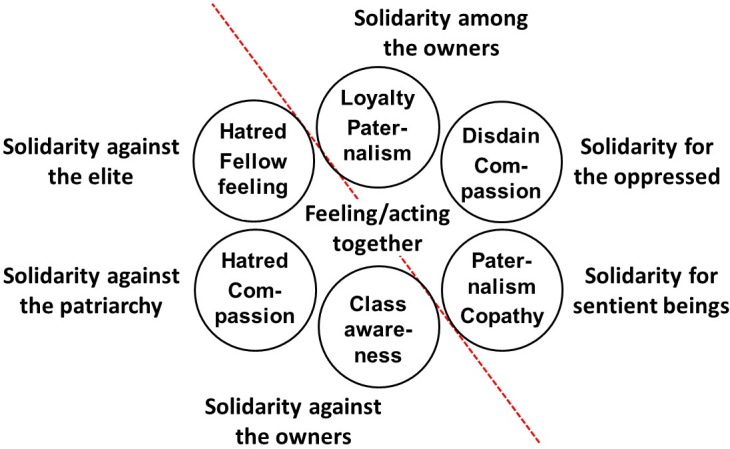
Solidarity as feeling and acting together dispersed on the map of justice.

Solidarity in the broad sense of feeling and acting together can be placed anywhere on the map, as shown in Figure 2.

The wider definition initially rescues some cases of corona solidarity. We only need to concentrate on the upper right half of the map (separated by the dotted line). Utilitarians can nurture a fellow feeling toward all sentient beings; capability theorists root for those who, due to oppression, have preferences that do not benefit them; and perhaps libertarians can assume some kind of a siblinghood among those whose property they see in need of protection.

In the light of these, businesses that protect themselves and others in their production chain against economic peril express solidarity in the libertarian sense and can extend their benevolence, by paternalistic measures, to their clientele. Who could live without Starbucks? Government actions that aid businesses contribute to these indirectly. Capability theorists, who slightly disdain the uneducated masses for not committing themselves wholeheartedly to the public health effort, can claim that compassion for the ignorant motivates their support of the same business and political practices. And since utilitarians can sympathize equally with all living beings capable of experiencing pain and pleasure, they can extend their fellow feeling, and justify solidaristic interventions, as widely as needed for the happiness of the greatest number.

The capability and utilitarian responses show the weaknesses of widening the scope of solidarity like this. Their benevolence and altruism are not in question, but they are universalist top-down approaches that do not naturally leave room for a we feeling of acting together for a common cause. The we feeling can be found, but especially in utilitarianism it is a very thin form of sympathy that I have called *copathy*—“a calm sensation or realization that we are one with all other sentient beings, and that we should not by our actions or choices make their lot worse” (see note 17). The distinction between “us” and “them” is in evidence, but somehow in a wrong sense. We are doing something that they (the ignorant and nonhuman animals) are not. Although fully fledged reciprocity is probably not a sensible requirement, some give and take, even in principle, would seem to be a part of proper solidarity.

## Back to Partiality—The Good, The Bad, and The Ugly

On the lower left half of Figure 2, we find the theories of justice, and accounts of solidarity, that build the solidaristic we feeling by naming an opponent—a culprit against which “we” must rise.

Early demands of salary equality for care workers and improvements to the working conditions of all those in the front lines heralded the temporary return of social-democratic displays of solidarity within professions and vocations and between them. In some countries, the uprising was resolved by local pay rises in the free-market spirit of prices going up with increased demand; in others, the struggle continues.^18^ Crossing the ideological border between positionalism and universalism (or the dotted line in Figure 2), utilitarians and capability theorists can join this battle of good (corona) solidarity with the realization that the majority of us are held in a precarious position by global capitalism and its agents.

Care and recognition ethicists in the bottom left corner of Figures 1 and 2 can also join, but with notable caveats. They originally share a (feminist) compassion with the capability ethicists,^19^^,^^20^ but the message has, of late, become more concentrated on the recognition of intersectional discrimination and oppression. As the COVID-19 pandemic has provoked xenophobic and racist incidents on all continents,^21^ displays of solidarity within the ethics of identity and recognition have become physical confrontations in the form of antiracist demonstrations.^22^

In the meantime, parochial communitarians in the top left corner of the map have risen against what they see as the elites restricting people’s lives unnecessarily in the name of the pandemic, which some of them believe is directly and intentionally caused by those in power or by hidden groups that secretly run the world.^23^ Antiregulation protests all over the world testify to the practical allure of this kind of thinking. The result is that the collectivist views in Figures 1 and 2, communitarianism on the one hand and care and recognition ethics on the other, are quite literally at each other’s throats, fueled by mutual hatred, apparently oblivious to the fact that they could share the same enemy with socialists, namely global capitalism.

The clearest examples of solidarity narrowly construed can, then, be found to the left of the dotted line in Figure 2. The reason why “corona solidarity” has terminological appeal is not, however, that protesters on both sides of the parochial-progressive divide clearly belong to groups with common interests or ideologies. It is rather that the expressions of workers’ solidarity could well be supported by utilitarians and capability ethicists. The latter’s recommendations are solidaristic only in a conceptually wobbly sense, but that does not seem to trouble those who include capability and welfare promotion in the sphere of solidarity. One way forward could be to build on that, learn to know our theories^24^ and learn how to communicate them so that they do not cause riots.^25^

## Notes

1. Broom D. A pandemic of solidarity? This is how people are supporting one another as coronavirus spreads. *World Economic Forum* 16 Mar 2020; available at https://www.weforum.org/agenda/2020/03/covid-19-coronavirus-solidarity-help-pandemic/

2. Sánchez Nicolás E. Coronavirus crisis deepens, but solidarity blooms. *EUobserver* 30 Mar 2020; available at https://euobserver.com/coronavirus/147917

3. Häyry M. European values in bioethics: Why, what, and how to be used? Theoretical Medicine and Bioethics 2003;24:199–214; available at https://www.researchgate.net/publication/10589587_European_Values_in_Bioethics_Why_What_and_How_to_be_Used1294804610.1023/a:1024814710487

4. Häyry M. Precaution and solidarity. Cambridge Quarterly of Healthcare Ethics 2005;14:199–206; available at https://www.researchgate.net/publication/7853088_Precaution_and_Solidarity1588781210.1017/s0963180105050255

5. Häyry M. Causation, responsibility, and harm: How the discursive shift from law and ethics to social justice sealed the plight of nonhuman animals. Cambridge Quarterly of Healthcare Ethics 2020;29:246–67; available at https://www.researchgate.net/publication/339856428_Causation_Responsibility_and_Harm_How_the_Discursive_Shift_from_Law_and_Ethics_to_Social_Justice_Sealed_the_Plight_of_Nonhuman_ Animals3215948810.1017/S096318011900104X

6. Häyry M. Doctrines and dimensions of justice: Their historical backgrounds and ideological underpinnings. Cambridge Quarterly of Healthcare Ethics 2018;27:188–216; available at https://acris.aalto.fi/ws/portalfiles/portal/29745112/H_yry_2018a_Last_Submission.pdf2950911910.1017/S096318011700055X

7. Houtepen R, ter Meulen R. Solidarity in health care. Health Care Analysis 2000;8:329–411.1115555510.1023/A:1026530912872

8. Kolers A. Solidarity and similarity: What kind of “we” are we, and why does it matter? *Journal of Medical Ethics Blog* 21 July 2020; available at https://blogs.bmj.com/medical-ethics/2020/07/21/solidarity-and-similarity-what-kind-of-we-are-we-and-why-does-it-matter/

9. Riordan D, Hoffstaedter G, Robinson R, Pryor E. Delivery workers are now essential. They deserve the rights of other employees. *The Conversation* 29 Mar 2020; available at https://theconversation.com/delivery-workers-are-now-essential-they-deserve-the-rights-of-other-employees-134406

10. *Starbucks’ Stories & News* 4 and 6 Mar 2020; available at https://stories.starbucks.com/stories/2020/navigating-through-covid-19/

11. Williams R. Letter to partners: Caring for Starbucks partners during COVID-19. *Starbucks’ Stories & News* 11 March 2020. Available: https://stories.starbucks.com/press/2020/letter-to-partners-caring-for-starbucks-partners-during-covid-19/

12. Russia helps Italy to fight coronavirus without any strings attached—Kremlin. *TASS* 23 Mar 2020; available at https://tass.com/politics/1133967

13. China offers help to 150 countries, 4 int’l organizations to fight COVID-19: White paper. *^Xinhua^* 7 June 2020; available at http://www.xinhuanet.com/english/2020-06/07/c_139120830.htm

14. The hidden world of the doctors Cuba sends overseas. *BBC News* 14 May 2019; available at https://www.bbc.com/news/uk-48214513

15. Sullivan A. Unmasking the EU’s coronavirus recovery fund—the fine print. *DW* 21 July 2020.

16. Häyry M, Takala T. American principles, European values, and the mezzanine rules of ethical genetic data banking Häyry M, Chadwick R, Árnason V, Árnason G, eds. The Ethics and Governance of Human Genetic Databases: European Perspectives. Cambridge: Cambridge University Press; 2007:14–36.

17. Häyry M. Hyötyä tavoitteleva ihminen (The human being as a utility seeker, in Finnish). Hänninen V, Aaltola E, eds. *Ihminen kaleidoskoopissa: Ihmiskäsitysten kirjoa tutkimassa* (The human being in a kaleidoscope: Examining the diversity of notions of humanity). Helsinki: Gaudeamus, 2020:105–127.

18. Busby M. Protesters march for fair pay for nurses and other NHS staff. *The Guardian* 8 Aug 2020; available at https://www.theguardian.com/society/2020/aug/08/hundreds-march-fair-pay-nhs-nurses-coronavirus

19. Gilligan C. In a Different Voice: Psychological Theory and Women’s Development. Cambridge, MA: Harvard University Press; 1982.

20. Nussbaum M. Sex and Social Justice. Oxford: Oxford University Press; 1998.

21. List of incidents of xenophobia and racism related to the COVID-19 pandemic. *Wikipedia*; available at https://en.wikipedia.org/wiki/List_of_incidents_of_xenophobia_and_racism_related_to_the_COVID-19_pandemic

22. Jha A. Why protests aren’t as dangerous for spreading coronavirus as you might think. *The Guardian* 18 Jun 2020; available at https://www.theguardian.com/commentisfree/2020/jun/18/anti-racism-protests-coronavirus-rise-covid-19-cases

23. Protests over responses to the COVID-19 pandemic. *Wikipedia*; available at https://en.wikipedia.org/wiki/Protests_over_responses_to_the_COVID-19_pandemic

24. Häyry M. The COVID-19 pandemic: A month of bioethics in Finland. Cambridge Quarterly of Healthcare Ethics 2020:1–9. doi:10.1017/S0963180120000432; available at https://www.cambridge.org/core/services/aop-cambridge-core/content/view/AAB9DFABFEA34D8F0AF6E313B6D994BC/S0963180120000432a.pdf/covid19_pandemic_a_month_of_bioethics_in_finland.pdfPMC721818232349824

25. Häyry M. The COVID-19 pandemic: Healthcare crisis leadership as ethics communication. Cambridge Quarterly of Healthcare Ethics 2020:1–14. doi:10.1017/S0963180120000444; available at https://www.cambridge.org/core/services/aop-cambridge-core/content/view/CE6B85449991BF962CF232B2FC9CB9C1/S0963180120000444a.pdf/covid19_pandemic_healthcare_crisis_leadership_as_ethics_communication.pdfPMC728729832438949

