# COVID-19: Another Look at Solidarity—ADDENDUM

**DOI:** 10.1017/S0963180122000068

**Published:** 2022-04

**Authors:** Matti Häyry

The standard boxed text for the “Dissecting Bioethetics” section of *Cambridge Quarterly of Healthcare Ethics* was omitted from the original online version of this article.^1^ The boxed text reads as follows:

“Dissecting Bioethics” welcomes contributions on the conceptual and theoretical dimensions of bioethics. The department is dedicated to the idea that words defined by bioethicists and others should not be allowed to imprison people’s actual concerns, emotions, and thoughts. Papers that expose the many meanings of a concept, describe the different readings of a moral doctrine, or provide an alternative angle to seemingly self-evident issues are particularly appreciated. To submit a paper or to discuss a suitable topic, contact Matti Häyry at matti.hayry@aalto.fi and Tuija Takala at tuija.takala@helsinki.fi.

## Note

1.

Häyry M. COVID-19: Another Look at Solidarity. Cambridge Quarterly of Healthcare Ethics 2022;31(2): 256–62. 10.1017/S0963180120001115.33342454PMC7844211

